# Part III: Recovery-Oriented Practices in Community Mental Health and Substance Abuse Services: A Meta-Synthesis

**DOI:** 10.3390/ijerph182413180

**Published:** 2021-12-14

**Authors:** Trude Klevan, Mona Sommer, Marit Borg, Bengt Karlsson, Rolf Sundet, Hesook Suzie Kim

**Affiliations:** Center for Mental Health and Substance Abuse, Department of Health, Social and Welfare Studies, Faculty of Health and Social Sciences, University of South-Eastern Norway (USN), 3040 Drammen, Norway; marit.borg@usn.no (M.B.); bengt.karlsson@usn.no (B.K.); rolf.sundet@usn.no (R.S.); hsuziekim@comcast.net (H.S.K.)

**Keywords:** recovery, meta-synthesis, mental health and substance abuse, recovery-oriented services, recovery-oriented practice, recovery capital

## Abstract

In recent decades, recovery-oriented practice has become the major approach in mental health and substance abuse care, especially in community mental health and substance abuse services. Various models of recovery-oriented practice have come to form the basis of the integration of this approach in service settings. The study aims to elucidate the characteristics of recovery-oriented practice as experienced by participants in the practice. The method used was a qualitative meta-synthesis that integrated the findings from thirty-four empirical papers published by one research group. Four meta-themes were developed: (a) helping and supporting, (b) collaborating and relating, (c) identity integration in practice, and (d) generating hope through nurturing and helping. These themes emphasize the value of relationships and connectedness, contextuality, and resources that can be mobilized in practice. The results emphasize the need to incorporate the elements in the four major themes as “working capital” for practitioners to realize recovery-oriented practice. The concepts of personal, social, and economic capital as working capital are elaborated, drawing from the meta-themes as the basis for recovery-oriented practice in mental health and substance abuse services.

## 1. Introduction

This is Paper 3 in a series based on meta-syntheses of the characteristics of recovery and recovery-oriented practices studied at the Center for Mental Health and Substance Abuse (CMHSA) of the University of South-Eastern Norway. Recovery has been a key area of research at the Center for more than two decades. The many studies conducted by the CMHSA over the years encompass a variety of analyses, descriptions, interpretations, and suggestions as to how “recovery” and “recovery-oriented practices” can be meaningfully understood and practiced. The studies draw on subjective experiences of living and dealing with mental health and substance abuse problems, and experiences of family members and professionals. Paper 1 and Paper 2 presented the results of meta-syntheses regarding experiences of recovery and processes of recovery [1,2]. While Paper I and Paper 2 focused on exploring the nature and characteristics of experiences and processes of recovery, the meta-synthesis presented in this paper (Paper 3) focuses on exploring recovery-oriented practices in community mental health and substance abuse (MHSA) services. The research question addressed is: How are recovery-oriented practices experienced and described in community MHSA service settings?

With the international movement towards recovery-oriented MHSA services, there has been an increased focus on developing and describing recovery-oriented practices [3]. Recovery-oriented practices have been described and presented in a variety of ways and in various contexts. In this paper, when using the plural term practices, we refer to “the actual doing of things” in terms of actions and collaboration on a daily basis in the context of community MHSA services. In general, the vision of recovery-oriented practice is considered to be: “(…) the aftermath of the era of deinstitutionalization” [4] (p. 11). Recovery-oriented practice in the community also draws on the work and learnings of Basaglia and the Italian democratic mental health reform [5]. Recovery-oriented support is about enabling people who have been placed at the margins of society to reclaim their basic citizenship as free and autonomous actors [6]. As Rowe suggests, rather than recovery being a precondition of citizenship, it is achieved through citizenship [7]. This is later followed up by voicing the need to ‘recover citizenship’, through orientation to the five Rs: rights, responsibilities, roles, resources and relationships. In a democratic society these five Rs need to be made available to its members through public and social institutions [8]. Recovering citizenships resonates well with the reports from UN Special Rapporteur claiming the right of everyone to the enjoyment of the highest attainable standard of physical and mental health [9,10].

With this orientation, the major voice has been the calls for a paradigm shift from the standard individually based biomedical and manualized services [11,12,13]). However, there are developments of recovery-oriented services without the fundamental ideological shift away from the biomedical orientation, in which the concept of recovery continues to be tied to the notion of illness and pathology. Illness management and recovery is an example of this [14]. The perspective of this paper and the research program at the CMHSA is rooted in the concept of recovery as tied intimately with everyday living, with the view that lived experience-based research is a critical knowledge base [15,16,17,18] and thus an important contribution to the movement toward this paradigm shift.

Recovery-oriented services are typically viewed in relation to the following key principles:

1. Relational recovery, emphasizing relationships and connectedness with people in a variety of social contexts. The recovery process is seen as inseparable from the social and cultural milieus of the people concerned [12]. The opportunity to choose coupled with tailored support to make well-informed decisions, and nurturing and maintaining hope, are also key factors [19].
2. Facilitation of peer support and new professional roles in services. The necessary organizational change encompasses new learnings and new practices oriented in reciprocal partnerships, people’s social life, social inclusion, and human rights [15,20]. As Perkins and Slade (p. 33) state: “Recovery focused services must start by considering not ‘the patient in our services’, but the ‘person in their life’, with a primary goal of helping people to live the life they want and do the things they want to do” [18].
3. A primary focus on human rights, living conditions, and social inclusion means treating service users as fellow human beings with the rights and obligations of people in general [21].
4. Community recovery, meaning that both the service context and the local community need support and development [13,15,19]. This means developing a new knowledge base and new professional skills oriented away from deficits and patient roles and towards everyday life issues, community life, employment, and rights to a safe home and sound finances.

Based on these four key principles, it can be argued that recovery-oriented MHSA services and practices are also entangled with community work and practices, understanding MHSA problems as contextual issues that are best understood and supported in the community.

Recovery-oriented practices in the community can be described as opportunities and activities for citizens in the community that are accessible for all [13,17,22,23]. Recovery orientation is about making sure that people with MHSA issues have the same opportunities, choices, and rights as everybody else. Taking back control over one’s life and experiencing citizenship are essential parts of recovery. Being seen as a valued citizen means access to housing, education and employment, ordinary community activities, and physical healthcare. For many people, tailored support is needed to promote this kind of community inclusion [11,19,21]. The literature on social recovery and recovery capital provides an organizational framework for such community inclusion [13,21,23]. The root of social recovery goes back to Warner who defined it as economic and residential independence with low social disruption [24]. Today, social recovery is concerned with people’s ability to lead meaningful and contributing lives as active citizens while experiencing mental health problems [13]). Social recovery focuses on community living and people’s resources and opportunities as opposed to a diagnosis, deviance, and service framework. Enabling people to participate fully in life means access and availability of resources and opportunities in the community. The concept of recovery capital provides a contextual way of mapping people’s existing strengths and resources and brings a focus on what needs to be done [23]. Tew describes five types of capital relevant for recovery and community inclusion: economic (money at one’s disposal), social (resources in one’s social network), identity (relations with significant others), personal or mental capital (coping and ways of seeing oneself) and relationship capital (the quality of close relationships) [23]. Thus, recovery capital challenges understandings of mental distress and recovery that individualize personal pathology and personal responsibility, suggesting the need for recovery-oriented practices to be attentive to personal, practical, relational, and social contexts.

Since the integration and incorporation of the philosophy and tenets of recovery and recovery-oriented practices in MHSA services, the past two decades have seen a huge rise in the number of scholarly and research publications in the field. This has also encouraged the publication of systematic reviews examining the status of the literature in this growing field [3,25,26,27,28,29,30,31,32,33,34,35,36,37,38,39]. This literature suggests that while recovery orientation has become firmly established in the culture of MHSA care and there is a consensus on the critical elements of recovery conceptualizations and the principles of recovery-oriented practice, there are still some controversies regarding three significant issues that have been voiced in the more recent literature [36,40].: (a) recovery as an outcome versus recovery as a process, which has implications regarding how the recovery perspective is integrated into practice, (b) a need for a comprehensive specification of the key components/characteristics/dimensions of recovery that embraces the complexity existing in human lives such as context, resources, and experiences of difficulties, and (c) the meaning and ramifications of recovery orientation in the context of the given social structure of mental health services with their historical and epistemological grounding in biomedicine and the focus on treatments, which influence the development of recovery-oriented services.

With our positioning and understanding of recovery as processes that are part of everyday life and contexts, often supported by professionals in the local community, this paper aims to provide a consolidated picture of recovery-oriented practices in the context of MHSA services. Through a qualitative meta-synthesis based on studies conducted in the Norwegian context, we aim to contribute to the knowledge base on how recovery-oriented practices can be described and understood, and how such practices may be perceived as entangled with local contexts and resources. This knowledge is important in the further development of context-sensitive, recovery-oriented MHSA services in the community.

## 2. Method

### 2.1. The Research Context

Recovery has been a key area of research at the CMHSA since the early 2000s. The Center has a specific focus on collaborative research methodologies with people with lived experience, family members, and practitioners. The CMHSA engages people with a variety of experiences and a wide range of knowledge as key partners in research. Our recovery research has from the outset focused on subjective experiences, relational aspects, everyday life experiences, and the impact of material and social conditions as well as recovery-oriented services, community development, and peer support work. Furthermore, the Center conducts research in dialogical and collaborative practices, and child and adolescent issues. The researchers have varied professional backgrounds in the health and social care sector and a wide range of experience of clinical practice, in addition to lived experience. The Center has expertise in qualitative, quantitative, and triangulation/mixed methodologies.

### 2.2. Qualitative Meta-Syntheses

The method applied in this paper is a form of qualitative meta-synthesis, and the procedures described in the following are equal to the chosen method in the Part I and Part II papers [1,2]. ‘Qualitative meta-synthesis’ as a method refers to a variety of approaches and is often used in systematic review studies. The qualitative meta-synthesis in this paper is in line with the first kind of synthesis identified by Sandelowski, Docherty, and Emden, which referred to integrating the findings from multiple qualitative studies within a program of research by the same investigators [41]. The purpose of this approach in the present paper is to explore how recovery-oriented practice is described in empirical research at the CMHSA, addressing the research question: “How is recovery-oriented practice described in empirical research at the CMHSA in the period 2005–2020?” The objective is to arrive at a theoretically meaningful synthesis of recovery-oriented practice as experiences and processes through the integration and comparison of the qualitative empirical data accumulated by CMHSA researchers in their studies of community MHSA practices. The procedural steps adopted reflect the seven steps identified by Noblit and Hare for meta-ethnography, which consist of (1) getting started, (2) deciding what is relevant to the initial interest, (3) reading the studies, (4) determining how the studies are related, (5) translating the studies into one another, (6) synthesizing translations, and (7) expressing the synthesis [42].

The publications included in this meta-synthesis were written by CMHSA researchers, whose research orientation as a group is recovery and recovery-oriented practice. The focus of this synthesis was recovery-oriented mental health and substance abuse practices, following up on the syntheses carried out for Parts 1 and 2 of this series of meta-synthesis papers. The first four steps of Noblit and Hare’s method have been well established within the group. This qualitative meta-synthesis thus encompasses the last three steps, namely translating the studies into one another, synthesizing those translations, and expressing the synthesis. Meta-ethnography and meta-syntheses in general are oriented towards “synthesizing” researchers’ interpretations of qualitative data in original studies, which are social constructions “built into accounts of methods, in the theories used, in the researchers’ worldviews” [42] (p. 3). However, this meta-synthesis did not have to deal with the issue of synthesizing different perspectives or worldviews. It began with the prior knowledge of our perspectives, methods, and worldviews, which align with the epistemological stance of a phenomenological-interpretive and critical perspective. For the fifth step of translating the studies into one another, the themes and concepts from each study with their descriptors were identified, compared, and contrasted, which also involved reflections on the relevant literature. Based on the results from the fifth step, the sixth step involved meta-synthesizing the themes and concepts regarding recovery experiences, processes, and practice orientations. This step thus involved using the researchers’ judgment and creativity, which is critical in qualitative synthesis [43]. The synthesis of themes and concepts found in these publications involved grouping similar themes together and specifying them into meta-themes by comparing the themes and their meanings. Some themes extracted from individual publications were also specified as meta-themes if they were considered critical in providing the meanings of recovery experiences, processes, or practice orientations. The seventh step of the meta-synthesis, “expressing the synthesis”, involved systematizing the results of the meta-synthesis.

**Figure 1:**
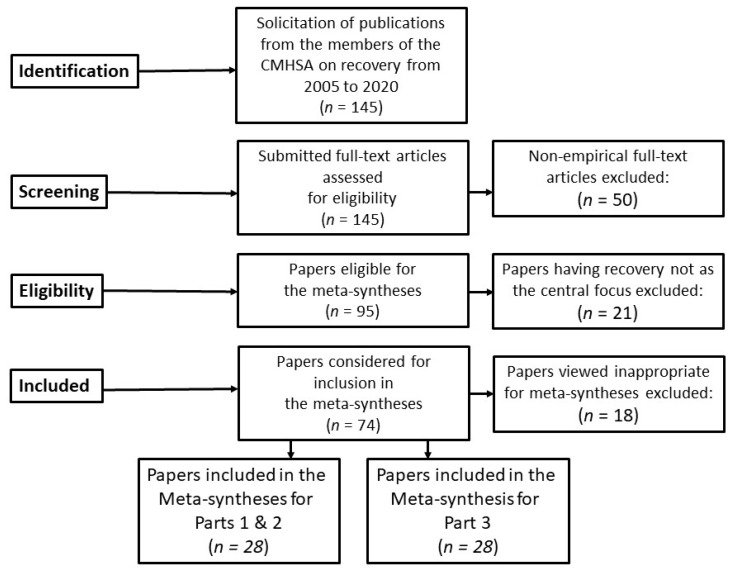
The steps taken by the research team for the meta-syntheses for Parts 1, 2, and 3.

Figure 1 shows the steps taken by the research team for the meta-syntheses for Parts 1, 2, and 3, using a PRISMA flow diagram. The details of the steps followed in assembling the database for this study are somewhat simplified because the publications included in these meta-syntheses were those of the members of the CMHSA research team.

The steps of collecting, reviewing, and analyzing the papers were as follows. A core research group of five CMHSA researchers was established to be responsible for the meta-syntheses and writing the results for publication. All 20 researchers in CMHSA were then invited to contribute to the study and requested to submit their publications to the core group. Sixteen researchers accepted the invitation. The inclusion criteria for the publications were empirical papers published from 2005 to 2020 with a focus on recovery as personal, social, and relational experiences and processes and on recovery-oriented services. We also invited the researchers to include other papers that might be relevant to the topic. The languages included were English and Scandinavian languages (Norwegian, Danish, and Swedish). A total of 145 papers were submitted.

These papers were reviewed by the core research group in relation to the research questions, resulting in the final selection of 95 empirically oriented papers. Each of these papers was systematized by using a data extraction form inspired by the Critical Appraisal Skills Program (CASP) for quality appraisal in qualitative evidence synthesis [44]. These studies employed qualitative methods, mostly focus group and in-depth individual interviews with research participants who were service users, family members or significant others of service users, and professionals. The analytical methods used in these studies were descriptive and/or interpretive. An examination of this set of publications by the core group resulted in a division of the material into three broad topic areas: (a) recovery as personal and/or contextual experiences, (b) recovery as processual, and (c) recovery-oriented services and practice. Therefore, three meta-syntheses were performed using these data. Of the set of 74 papers judged to be appropriate for inclusion in the three meta-syntheses planned, there were 28 papers in the areas of recovery as personal and/or contextual experiences and as processual, of which two papers of meta-syntheses were published [1,2], and 46 papers in the area of recovery-oriented services and practices. For the current meta-synthesis, we reviewed these 46 papers carefully and only retained 28 papers for the meta-synthesis. The major reasons for the exclusion of 18 papers were that the papers did not focus on recovery-oriented services and practice, were policy-oriented or philosophical or that they did not deal with experiential knowledge about experiences of recovery-oriented practices.

## 3. Results

The themes presented in this results section were developed by collating and synthesizing similar themes in the included papers. While the synthesis aims to capture overarching patterns and themes across the included papers, we also aim to present how experiences of recovery-oriented practices are multifaceted and involve diversity and variety. Experiences of recovery-oriented practices are represented by four major themes: (a) helping and supporting, (b) collaborating and relating, (c) identity integration in practice, and (d) generating hope through nurturing and helping. These themes emerged as representing the experiences of recovery-oriented practices in MHSA services from the perspectives of service users, family caregivers, and professionals/service providers.

### 3.1. Helping and Supporting

The theme of *helping and supporting* refers to the vital elements of recovery-oriented practices crucial for the intended help and support to be actually experienced as helpful and supportive. It encompasses five sub-themes: (a) being helped on one’s own terms, (b) timely helping, (c) creative and collaborative helping and supporting, (d) helpful actions, and (e) helping for different needs (shown in Appendix A Table A1).

#### 3.1.1. Being Helped on One’s Own Terms

It is essential to be helped on one’s own terms and to be in charge of one’s own life [45,46]. To be helped on one’s own terms also includes identifying the person’s strengths and resources [47]. It is crucial to be involved in a collaborative exploration of the person’s own solutions to everyday challenges and to negotiate solutions the person can live with [47]. Supporting and facilitating work integration and meaningful activities based on the service user’s own preferences and resources were often threatened by the professionals’ lack of belief in the service user’s potential [48] (Kinn et al., 2016). Furthermore, professionals expressed skepticism towards staff expertise in vocational programs, based on experiences that service users received insufficient job support [48]. Thus, valuing service users’ interests and needs by discovering the activities they find meaningful and those that improve their self-esteem and confidence was important for the help to be experienced as helpful.

#### 3.1.2. Timely Helping

Timely helping is emphasized as an important aspect of recovery-oriented practices and means giving the right help at the right time. Help and support need to be sensitive to the person’s own process and day to day state of health. Some days require fewer demands. When doing the dishes feels like too much, it is helpful to be supported in ‘taking one cup at a time’ [49]. Professionals being available when help is needed was described as an important aspect of help and support in recovery-oriented practices [45,50]. Long waiting times for services often exacerbate problems, and timeliness was greatly appreciated [50]. Timely helping also included seizing the right moment to help. This requires a sensitive presence and awareness of what the particular situation calls for [51]. For the help and support to be truly helpful and supportive, assuring continuity is highly valued. To be there over time, for as long as needed, strengthened the relationship between the practitioner and the service user and was fundamental for help to be experienced as helpful and supportive [52].

#### 3.1.3. Creative and Collaborative Helping and Supporting

Creative and collaborative help and support is considered important to recovery-oriented practices. Klevan and colleagues found that helpful help was experienced as something creative and ‘in the making’ [53]. This is in contrast to a predetermined and defined approach to helping and supporting. Creating new and different ways of helping and supporting through collaboration between practitioners and service users that challenged traditional roles and relationships, opened up new approaches felt to be more fruitful for recovery [53]. Creative and collaborative help and support had the potential to address dilemmas due to service users’ life challenges and problematic structural factors in recovery-oriented practices [54]. Help and support as a co-creative process requires knowledge and flexibility to balance different interests and needs [52]).

#### 3.1.4. Helpful Actions

Helpful actions are actions that facilitate recovery processes and are highlighted as fundamental for recovery-oriented practices. Helpful conversations encourage action and enable service users to think aloud about their everyday situation and to put words to feelings [47,55]. This helps to raise awareness and solve problems. Andvig and Biong found that helpful conversations could be about everyday issues, but could also be about deeper and more existential topics, such as politics and religion, when the service users initiated these topics [47]. On the other hand, it is also important to have an awareness of service users’ condition and to try to protect them from topics that could be harmful if they are going through a difficult period [47]. Helpful actions created a change in momentum from stagnation to action [56]. Actions aimed at eliminating barriers to services and increasing participation in the community provided service users with hope for the future and were of great importance in their recovery processes [57].

#### 3.1.5. Helping for Different Needs

Help and support in recovery-oriented practices are directed towards different needs. Providing practical help, such as organizing one’s everyday life including work, housing, and family life, was helpful when a crisis threatened daily life structures [49,58,59]. Collaborating with the local community and supporting service users in familiarizing themselves with the local environments was emphasized by practitioners [46]. Helping to empower and to uphold self-worth was of importance in preventing a feeling of losing one’s sense of self as normal when a mental health crisis occurred and normal everyday life collapsed [49]. Maintaining the feeling of safety by being present and accessible even at nighttime was an important aspect of help and support in times of crisis [49]. Helping to increase service users’ knowledge by informing them about what will happen when they receive mental health services is crucial in preventing feelings of confusion and insecurity and in improving experiences of continuity of care [50].

### 3.2. Collaborating and Relating

Collaborating and relating refer to supportive interpersonal aspects and the supportive characteristics of professionals in collaborative relationships with service users in recovery-oriented practices. They also include organizational conditions and strategies that promote recovery-oriented practices. Three sub-themes were identified: (a) relational characteristics, (b) characteristics of professionals in collaborative relationships, and (c) organizational conditions and strategies.

#### 3.2.1. Relational Characteristics

Trust is at the core of interpersonal support and is a prerequisite for recovery-oriented practices [47,50,55,60]. Trusting the other to take one’s vulnerability into account is of importance for feeling safe and laying the foundation for a mutual and collaborative relationship [58,60]. Sommer and colleagues found that young people struggling with mental health problems greatly appreciated relationships that made them feel accepted and welcomed as significant to the other [60]. “Felt togetherness” included feeling an attuned resonance and connection with the other and had the potential to evolve when service users and professionals shared personal experiences or did something together [58,60]. Recovery-oriented relationships uphold mutuality and the possibility to flourish in joint participation through mutual respect and mutual disclosure [60]. Mutuality includes welcoming service user involvement and sorting things out together, where both professionals and service users take the initiative [45,46,50,58,60,61].

#### 3.2.2. Characteristics of Professionals in Collaborative Relationships

Many qualities were emphasized as significant for professionals in collaboration with service users. Being respectful and non-judgmental [58], trusting and safe [55,62]), caring and available [45], and not being a distanced professional [55] reflect crucial qualities in recovery-oriented practices. Further, being flexible, such as being able to help with service users’ needs as needed, was highlighted as important [46]. Flexibility also includes having the freedom to assess and decide on the actions required to meet service users’ needs [46]. Being a resource and being an advocate for service users were important to service users’ ability to access and comprehend services and the local community [46,62,63]. Skills in talking with service users about difficult topics, in involving family members appropriately, and in being able to tune into the moment and situation in spontaneous and informal ways were found to be important factors [51,63,64].

#### 3.2.3. Organizational Conditions and Strategies

Organizational conditions and strategies refer to how the recovery-oriented organizing of services and the conditions in which professionals work can facilitate and/or hinder recovery-oriented ways of collaborating and relating, and thus, the development of recovery-oriented practices.

Interprofessional collaboration is an important aspect of recovery-oriented practices within and between different services. Establishing organizational strategies that enhance communication skills, insight into the values and conditions necessary in decision making, and shared understandings are crucial to interprofessional collaboration [65]. Such collaboration also rests on practical issues, such as having routines and allocated time for meeting regularly. Organizational strategies that are recovery oriented also involve strategies that enable professionals to have access to key resources such as appropriate housing for service users, and to see and collaborate with the service user as a whole person [45]. Such organizational strategies need to be open-ended, in terms of allowing professionals a certain autonomy and leeway to work and collaborate in recovery-oriented ways. Rigid organization of services and lack of autonomy may hinder professionals in meeting their responsibilities and may thus also constrain the development of recovery-oriented ways of working and collaborating [46,66]. Furthermore, bureaucracy and neoliberal organization of services may hinder flexibility and determine how professionals relate to service users and their relatives. A combination of neoliberalism and medical logic as an organizing principle in services may challenge humanistic and recovery-oriented values and understandings of collaboration and may thus inhibit recovery-oriented practices [67,68]. Organizational strategies that can be perceived as recovery-oriented need to involve an overall organizational transformation in order to facilitate flexibility, continuity of care, and collaborative partnerships between professionals, and between professionals and service users [50,66].

### 3.3. Identity Integration in Practice

The theme identity integration in practice relates to how an important part of recovery-oriented services is to focus on and enable identity work, and is viewed as involving two sub-themes: (a) promotion of individual identity and (b) promotion of strength-based identity in service users.

#### 3.3.1. Promoting Individual Identity

An important part of promoting individual identity in recovery-oriented services is to develop practices that enable an awareness of how gendered understandings and stereotypes among healthcare professionals might have implications for how they interpret service users’ conditions and situations, and that involve professionals in working with service users on an individual or broader level in service provision and in organizational contexts [69]. Identity work in services involves the ability and possibility to think, work, and collaborate beyond the identity of “being mentally ill”, and to allow service users to hold multiple identities. This involves understanding identities as fluid and processual. Thus, identity work in services needs to focus on both the importance of being and becoming oneself and the discovery of new aspects of oneself and new experiences [70].

#### 3.3.2. Promoting Strength-Oriented Identity

Promoting strength-oriented identity involves recognizing service users as actors in their own lives and in shared decision-making processes. Interaction skills and sensitivity are prerequisites for developing trusting relationships that can enable recovery-oriented practices and conversations [49]. Through a focus on service users’ everyday lives and on life in general, their strengths and their own suggestions for solutions can be emphasized. Such dialogues can create hope and lead to action, and may be crucial in strength-based identity work and in developing recovery-oriented practices [47,55].

### 3.4. Generating Hope through Nurturing and Helping

Hope can be vital to recovery, and is therefore emphasized as an important element of recovery-oriented services. To generate hope through nurturing and helping involves practices that support service users in becoming hopeful and generating hope in the context of difficulties. Thus, two sub-themes are identified: (a) supporting service users to become hopeful, and (b) generating hope in the context of difficulties.

#### 3.4.1. Supporting Service Users to Become Hopeful

Receiving support to become hopeful is considered an important part of recovery processes and thus to support and nurture hope is considered a key aspect of mental health practitioners’ practice [71]. Supporting service users’ hope involves helping them to believe in themselves and others and helping them to see and acknowledge opportunities by pointing out that the future is open. Thus, hope is not something that merely “is”, but is perceived as a joint venture created through relational work and practices [47,71]. An important requirement for the ability to nurture others’ hope and to hope on behalf of others is the practitioner’s own hope. This hope is vital in being able to perform sometimes challenging work [71,72,73]. The importance of the practitioner’s hope needs to be acknowledged in services, and to be facilitated through practices that involve flexibility and openness at the organizational level [71,72]. Flexibility and non-bureaucracy in services are also considered to be pivotal by service users, as experiencing generosity and being treated as an individual with unique needs and resources may enhance hope as part of recovery processes [47,57].

#### 3.4.2. Generating Hope in the Context of Difficulties

Working with hope as part of recovery processes also involves generating hope in the context of hardship and difficulties. Part of this work can involve practices that support service users in overcoming the distancing, disempowerment, and de-individualization that they may experience in diverse contexts, both inside and outside services. Thus, generating hope in such contexts may involve helping with battles with bureaucracy and clearing the path to hope and hopefulness despite setbacks and failures, recognizing working with hope as a bumpy and non-linear process [57,71].

## 4. Discussion

In this paper, recovery-oriented services are explored and described based on the experiences of service users, family members, and practitioners. The results of this meta-synthesis elaborate on how recovery-oriented practices stand out as multifaceted, although with certain common characteristics: the value of relationships and connectedness, the centrality of experience-based knowledge, and recovery as connected to community participation. Our meta-synthesis provides deeper insights into these characteristics through the nature of helpful relationships and of collaboration, and the emphasis on hope, identity work, and organizational conditions. There is growing interest in future developments of recovery-oriented services with an explicit focus on social, economic, political, and cultural determinants [40]. This is in line with the United Nations Special Rapporteur’s call for a recovery- and community-based approach to mental health, promoting social inclusion, rights-based treatments, and psychosocial support [9,10]. This understanding of MHSA difficulties and helpful approaches aligns well with our ideas based on the results of this meta-synthesis. The concept of recovery capital offers an open framework for understanding the variety of internal and external resources needed to overcome MHSA issues [23,74,75]. It is well recognized that recovery capital provides actual and potential resources for persons in recovery. Furthermore, recovery capital as a framework addresses the complex interplay between people’s recovery and their social and cultural contexts [23,76] and reflects the complexity revealed in the results of this meta-synthesis. These results imply that collaboration between practitioners in MSHA services and service users, as well as between services, community settings, and health and social care systems, provides important contributions to recovery [2,77,78]. Based on this understanding of recovery as contextual and relational processes, we would argue that practitioners in MSHA services also need access to recovery capital to collaborate and develop recovery-oriented practices that promote social inclusion and citizenship. Focusing on how practitioners need access to recovery capital underscores how recovery is connected to relational and contextual processes and not just individual projects and responsibilities. Following Bjørlykhaug et al., services with a social approach to recovery should work with recovery capital at all levels [79]. Drawing on the results of this meta-synthesis and using Tew’s framework for recovery capital [23], we will now discuss how practitioners’ access to economic capital, identity and personal capital, and social and relationship capital may be perceived as important prerequisites for developing recovery-oriented practices.

### 4.1. Practitioners’ Access to Economic Capital

The current study elaborates on how access to economic capital is important in recovery-oriented practices. Receiving support to have enough money to live on and a place to live is emphasized as important, and accordingly services need to be organized in ways that provide professionals with access to adequate resources such as housing [47]. Partnerships with providers responsible for citizens’ general living conditions are also essential. Practitioners who emphasize creating collaborative networks of services are pivotal in recovery-oriented practices and in securing people’s basic human rights [75]. However, the fragmentation and specialization of services and the lack of a whole-system approach means that practitioners in MHSA often have limited access to economic capital. In the context of community services, addressing economic and practical needs are issues that are commonly allocated to other services than MHSA. These services may involve different knowledge bases and professions and may not be working in recovery-oriented ways [77,80].

Recognizing recovery as social and contextual processes connected to basic human rights suggests the need for practitioners and MHSA services to have access to economic capital. This might suggest the need for MHSA services to be part of a more coherent mental health and social care system with a shared value base, or what Mezzina refers to as ‘a whole-system, recovery-oriented approach to community mental health care’ [80]. Ignoring or downplaying the importance of economic capital in developing recovery-oriented practices may imply that recovery is mainly understood as the responsibility of the individual [40]. This could also mean that recovery and recovery-oriented practices are perceived as the responsibility of the individual practitioner rather than the wider community and its range of services. In this way, in line with the findings of this meta-synthesis, economic capital encompasses not only what is available to service users but also to practitioners’ access to resources and activities in the local community and the social system, extending outside services.

### 4.2. Practitioners’ Access to Identity Capital and Personal Capital

Identity capital and personal capital are the basic resources individuals possess that specify their capacity to perform activities and build relationships in everyday life. Tew (2013) argues how identities may be ascribed based on status, achieved based on social interactions and performances, and managed through striving for acceptance and influence. Such identities may serve as currency for social participation and inclusion, and in this way, identity capital may help to promote social capital. While it may seem evident that developing identity capital that allows for moving beyond an identity as “mentally ill” and “service user” is beneficial to the person’s recovery, it is also necessary for practitioners in MHSA services to expand their identity capital beyond that of being a professional expert in order to develop as recovery-oriented practitioners. The current study shows how professionals who are flexible and can work in spontaneous and informal ways are considered to be helpful. While such competencies may be perceived as recovery oriented, they may also challenge more traditional understandings and standard guidelines about competencies that promote influence and acceptance among other professionals. Recovery-oriented services may thus entail that practitioners are caught between expectations from themselves and others about adhering to professional identities in line with recovery-oriented values of collaboration, mutuality, and context sensitivity, and a more traditional expert identity, characterized by following certain rules and procedures and having clinically relevant knowledge [53,81]. A pervasiveness of economic agendas, managerialism, and standardized assessments and approaches in MHSA services may compromise practices that focus on human relationships, collaboration, and mutuality [82]. Thus, recovery-oriented practices in MHSA services require a thorough discussion and reorientation about what a recovery-oriented professional identity may involve and presuppose, preferably enabling professionals’ identities to be dynamic and evolving to embrace their responsibilities to support service users’ identity and personal capital.

Having access to and working on identity capital that may lead to open-ended practices that are perceived as recovery oriented by those involved is also closely connected to constructive ways of seeing oneself and of engaging with the world. The latter is considered key to having access to personal capital [23,83]. Personal capital involves having a broad-based repertoire of coping and problem-solving strategies that enable people to deal with challenges. In the current study, practitioners’ own hope is described as necessary to nurture other’s hope and to perform sometimes challenging work. In this way, hope can be considered an important part of practitioners’ personal capital. Recovery-oriented services thus need to facilitate practices that can build resilience and nurture practitioners’ own hope, and thereby personal capital, through flexibility and openness at an organizational level [71,72,84].

### 4.3. Practitioners’ Access to Social Capital and Relationship Capital

The current meta-synthesis shows that social contexts and supportive relationships are crucial aspects in supporting people’s recovery. Hence, recovery-oriented practices need to involve awareness of the importance of enhancing people’s social and relationship capital. Relationship capital involves having significant others who can be there for the person through various ups and downs and who offer recognition and acceptance [23]. Further, relationships that support reciprocity and provide opportunities to give as well as to receive can be crucial to support a person’s recovery [75]. In the light of recovery capital, the results of this meta-synthesis emphasize the need for practitioners to have flexibility and opportunities to engage in a person’s social and family contexts, and to intervene beyond individually focused therapeutic work. The UN Special Rapporteur underlines how current practices are still strongly influenced by individually focused biomedical models [9,10]. This biomedical influence represents a barrier for MHSA services and practitioners in taking a contextual and social approach to enhance relationship capital.

Perceiving recovery as contextual and social implies that practitioners are also part of social contexts and networks that affect their collaboration in interaction with others. The term social capital refers to the sum of resources, actual or virtual, that an individual or group has access to and that may be beneficial in terms of providing information, support, and options [23,74]. While the importance of social capital is commonly used to describe the importance of the social relations and resources people struggling with MHSA problems need to improve their lives, it can also be argued that practitioners depend on this kind of capital. The current meta-synthesis shows how recovery-oriented practices require knowledge and flexibility to meet and support different needs, including meaningful activities, paid work, educational participation, and recreation activities. The results support previous research in highlighting the fact that recovery does not occur in a vacuum, but most often benefits from supportive social contexts, including accessible services [40]. The findings question the emphasis on recovery as solely an individual journey of self-actualization for which the individual is responsible, and advocate a collective and community-based form of recovery. Enhancing service users’ recovery capital may involve building bridges between people recovering from mental health problems and networks and meeting places in mainstream society [80]. In this sense, the social capital of professionals is a vital resource for service users. In order to help service users enhance their social capital, practitioners are dependent on collaboration with the local community and other services and settings supportive of recovery. However, this requires the availability of non-stigmatizing and non-discriminating social settings.

## 5. Conclusions

This meta-synthesis identifies the need to incorporate the elements of the four major themes, helping and supporting, collaborating and relating, identity integration in practice, and generating hope through nurturing and helping, as working capital for practitioners to actualize recovery orientation in everyday practice. Recovery-oriented practices are intertwined with service users’ social and contextual factors in the community. It makes no sense that services and practitioners are expected to have recovery-oriented attitudes and approaches while they have little or no access to resources in the local community that provide recovery capital. Without a range of social contexts beneficial to service users’ recovery capital, practitioners will have difficulty in providing recovery-oriented services. Unless recovery-oriented elements are identified and cultivated on community and system levels, practitioners will be deprived of possibilities to strengthen the service users’ recovery capital. It could be argued that within a recovery-oriented system of care, all systems should be supportive of recovery [40], which may involve working to change social attitudes in mainstream society, both at local and societal levels [85].

The results of the current meta-synthesis expand on elements that have been identified in various recovery-oriented practice models and in systematic reviews by delving deeply into the experiences and meanings of recovery-oriented practices. While focusing on recovery capital as an actual and potential resource for practitioners and services may seem controversial, the framework may also be perceived as beneficial in exploring and showcasing the relational and contextual nature of recovery-oriented practices. An emphasis on mental health services’ access to recovery capital recognizes that recovery-oriented services need to collaborate with actors and stakeholders in the local community. Further research should address the relationship between mental health practitioners’ access to recovery capital and recovery-oriented practices, and how such relationships may contribute to developing services that are sensitive to how social determinants affect people’s lives and possibilities for recovery.

## Data Availability

All the included studies are in Table A1.

## Appendix A

**Table A1:** List of the empirical papers included in the meta-synthesis (in chronological order of publication).

Publications	Research Question (s)	Methods	Research Participants	Themes and Meanings
[56]	Hvordan beskrives håpefull praksis av ansatte i et ambulant akuttpsykiatrisk team, og hvordan kan denne praksisen forstås? How do professionals in a crisis resolution team describe hopeful practice, and how can this practice be understood?	Phenomenological-hermeneutic using multi stage focus group interviews.	Eight professionals	The over-arching theme of “inspire and facilitate” was explored through three themes: To get in position (To understand the service user through being open and open one’es senses to the service user and their situation, to build trust—sometimes working “outside the box”) To get the service user in motion (To undersand the service user and what is important to the person through talk and practical approaches) To support the motions of the service user (Convey support and belief in the service user’s ability to master the situation, in the use of time, and moving outside the box/breaking rules)
[51]	How do environmental staff experience collaboration between staff and residents and how can these experiences be understood?	Phenomenological-hermeneutic approach using focus group interviews_._	18 professionals	Themes for the experiences of collaboration: Staff’s knowledge, traits and experience The spontaneous and informal process Conditions staff are unable to influence
[61]	How can the low-threshold activities program «Step by Step», aimed at people with substance abuse problems contribute to meaning and be an arena for community and coping?	collaborative and participatory research approach using multi-stage focus groups interviews	15 activity providers in the “Step by Step” program	Personal and social developing activities, Continuity and stability in life, A meaningful alcohol- and drug free arena To be met with respect and dignity.
[64]	To explore the experiences of ACT-practitioners’ potential to support service-users’ citizenship.	Phenomenological-hermeneutic approach, using multi-stage focus group interviews	5 professionals in ACT	Deserving trust Having dialogues about life Working together in partnerships
[6]	How do health professionals describe recovery-oriented conversations with their patients in a milieu therapeutic setting?	Acton research with multistage focus groups using a qualitative content analysis as the method of analysis	15 Mental health care professionals	Prerequisites for conversation - Developing trust - Sensing the right moment for conversation - Having competence The focus of conversation - Identifying patients’ strengths - Stimulating action-oriented reflections - Exploring the patients’ own solutions - Describing feelings - Creating hope - Talking about life in general Different views on topics of conversation - To go as deep as possible To protect the patient
[65]	To describe and interpret interprofessional collaboration between healthcare professionals working at the district psychiatric centre (DPC) and employed in community mental health care (CMHC)	Collaborative approach using multi stage focus group interviews.	18 professionals	One main theme “development of interprofessional collaboration by means of organizational strategies and interactional styles” with three categories: Improved communication skills (Getting to know each other, development of a common professional understanding) Ddeveloping structures for coordination and responsibility (Having routines and regular meetings) Increased professional insight into the values and conditions necessary for decision-making (Increased user involvement, Interactional flexibility in decision-making and Equality and respect between DPC and CMHC)
[63]	To identify key characteristics of the ways in which mental health practitioners collaborate with service users and their families in practice.	Actions research using multi stage focus groups	10 professionals	Three main themes: Walking alongside through negotiated dialogues, (hopes, dreams and goals of the service user as a starting point, supporting everyday challenges) Maintaining human relationships (not give up on people, important with time and continuity, take user involvement seriously) Maneuvering relationships and services (balancing, requires knowledge and flexibility)
[73]	Develop knowledge about mental health professionals’ experiences of job satisfaction	phenomenological-hermeneutical, using semi-structural interviews	6 mental health professionals	Three main themes: Job satisfaction is important for doing a good job Having hope impacts the job satisfaction Having good working conditions promotes and maintain job satisfaction.
[45]	To explore, describe, and interpret participants’ experiences with partaking in the Housing First project for persons with dual mental health and/or substance abuse problems	Hermeneutic-phenomenological approach using in-depth individual interviews	12 adults with MH and/or SA problems	Two major themes and sub-themes under the overall understanding of seeking security with a professional person one has confidence in and getting a grip on one’s life again: Having an available professional companion—Caring professionals; Professionals who are available; Help on your own terms Taking the lead in one’s own life—Empowerment, Recovery (Improved quality of life and belief in the future)
[59].	How do persons with co-occurring problems experience recovery orientation in a local MHSA team?	Phenomenological using individual interviews	13 services users with MHSA problems	The experience of the participants with recovery orientation in the service expressed as “*Here, they get a grip on things and do something about it.”* Respect and equality in partnerships with team, accessibility and flexibility. Ability to be active in the collaboration and take initiatives Useful with conversations, being together, practical help at home, help with money and with other services Experiences of better mental health and less drug-use
[59]	To investigate how, and on what grounds, involvement of relatives is perceived in Danish psychiatry	Multisite field work with a discursive approach		The conceptualizations of the mentally ill as “weak” (vulnerable, needing help, unable to carry on by themselves, etc.) versus the conceptualization of the relative (significant others) as “normal,” and resources (Making of the strong significant other—Transformation for relatives to be co-therapist or quasi professional in the treatment of the mentally ill) Mentally ill—(a) “Responsibilization” of being weak and requiring treatment (help); (b) the mentally ill person’s autonomy as “oughtonomy”—The person is expected to consent to treatment and dependency Relatives (Significant others)—(a) “Responsibilization” of the relative as an agent of support to facilitate/assure psychiatric treatment of the mentally ill; (b) Psychoeducation of relatives—Support and facilitate their involvement through educating and transforming them into semi-professionals; and (c) Recognition of relatives in involvement as contributing to (and economizing in) the care/treatment of the mentally ill (Cost-effectiveness)
[66]	To elaborate on how the framework for home care services affects the services’ work with older people with mental health problems living at home. Research question: How do employees in home care services describe their experiences with meeting this group of elderly in their daily work?	Fieldwork, observation and qualitative interviews—both individual and focus group	40 health care professionals	Before and now—From holistic to fragmented care (explores how new organization of services) affecting the content, many professionals involved, lack of continuity, lack of control, lack of time, less time for relational work—especially unfortunate for elderly with mental health issues) Different strategies in daily work life (lack of flexibility—balancing between stretching/opposing decisions and adjusting) Experiences of commitment (home services feeling committed to care for a group that receives little help and attention elsewhere) Neglect and inadequacy (not enough time, resources, feeling burdened and alone with responsibility)
[71]	To explore first-person accounts of how practitioners nurture and inspire hope.	Action research using in-depth interviews	Eight professionals in MHSA services	Believing in oneself and others Seeing and acknowledging opportunities Maneuvering towards hope
[63]	The purpose of this paper is to describe parents’ experiences of collaboration with mental health practitioners.	Thematic analysis of multi-stage focus group discussions	10 parents of young adults with MHSA problems	Negotiating partnerships Incomprehensible services Being the young adult’s advocate
[50]	To explore services users’ experiences and perceptions of continuity of care within and across services relevant to personal recovery, to elicit which dimensions of continuity of care are most essential to service users, and to generate ideas for improving service users’ experiences of continuity of care.	Hermeneutic phenomenological approach, using in-depth individual interviews.	10 service users	Relationships -from experiencing frequent setbacks and anxiety due to breaks in relationships, to feeling safe in an on-going personal relationship. Timeliness—from experiencing frustrating waiting times with worsening of problems, to getting help when needed. Mutuality—from having a one-sided struggle, to a situation in which both professionals and service users take initiatives. Choice—from not having the opportunity to make practical arrangements within the context of one’s everyday life, to having an array of support options to choose from Knowledge—from feeling confused and insecure because one does not know what is happening, to feeling safe because one is informed about what is going to happen.
[49]	To describe and explore service users’ experiences of mental health crisis and what they experience as hopeful help from crisis resolution teams	Hermeneutic phenomenological approach with in-depth individual interviews	14 service users who have received CRT services for mental health crisis	Experiences of crisis: Loosing foothold (The loss of structures & daily life structure) Becoming smaller & smaller (The loss of self-worth) On the edge (Crisis as a matter of life and death) Experiences of helpful help: One cup at a time (Help as structure and practical support) Not having to be afraid (Help as safety) Someone valuable (Help as supporting self-worth
[58]	How do young adults service users with co-occurring mental health and substance abuse problems understand and describe collaborative practice with community mental health practitioners?	Hermeneutic phenomenological, collaborative and action-oriented approach, using in-depth individual interviews	7 young adult services users who had experiences of receiving services from mental health agencies and substance abuse agencies	Don’t fix me or judge me (Being respectful and being met as fellow human being) Not giving up (Being responsive, receptive, and hopeful and with a belief in them) Someone to sort issues out with (Trusting relationship of being helpful) Practical help (Providing practical help)
[70]	To explore the significance of participation in a music and theatre workshop in terms of people’s experiences of identity	Hermeneutic phenomenological approach using in-depth, conversational individual interviews	11 adults with long-term mental health problems	Becoming a whole person—Enabling the person to be oneself and to become oneself Being allowed to hold multiple identities—Going beyond the identity of a mental health patienthood, the possibility of re-negotiating one’s identity to represent oneself as a multicreative person without simultaneously connecting this to one’s identify as being mentally ill Exploring diverse perspectives—Discovering new aspects of oneself and opening up to new experiences, perspective, and interpretations
[46]	To explore, describe, and interpret how providers apply a harm reduction approach within a housing project focused on individuals who are homeless with co-morbid substance use and mental health problems.	Inductive approach, using multistage focus group interviews	5 professionals	Letting the service user sit in the driver’s seat” “We don’t follow service provision contracts, we do everything” “Collaborating with the local community
[54]	To explore and describe staff experiences with dilemmas in recovery-oriented practice to support people with co-occurring disorders.	Focus group interviews. Thematic analysis	8 professionals working in community team	Three dilemmas were described: Balancing mastery and helplessness Balancing directiveness and a non-judgmental attitude, Balancing total abstinence and the acceptance of substance use.
[48]	To explore the community mental health professionals’ views of their clients’ work potential and their understanding of local vocational rehabilitation programs.	Hermeneutic phenomenological approach using focus group interviews	21 MHSA professionals	Three main themes: Viewing service users as vulnerable and not ready for employment, with the discovery of their own lack of beliefs in clients’ vocational potential as a latent barrier The laying stepping stones by practitioners to everyday life activities, from which clients could be launched into the community and meet new role responsibilities Displaying skepticism toward the competence of staff in vocational rehabilitation programs.
[53]	To identify and explore how clinicians in CRTs construct discourses of helpful help	Focus group interviews with a discursive approach	8 focus groups with professionals in CRTs	Help as made (The creators of something new and different) Help as given (The representatives of the expert system)
[69]	To investigate how professionals’ articulations of depression are framed by signs of masculinity and femininity, and how these articulations inform service provision to patients with depression in clinical psychiatry.	Ethnographic appraoch, using interviews	29 nurses, 10 medical doctors and 6 psychologists	Gendered differentiations—Women are most often diagnosed with depression and offered psychiatric treatment, while men with atypical depression that manifested themselves through drug use and were most often excluded from psychiatric treatment.
[68]	Aim: To explore how, and under what conditions, professionals involve relatives in clinical practice. Research questions: Which signs of taken-for-granted values and ideas about the working of the field (doxa) appeared in the empirical material from the respective clinic? When and how do professionals articulate their reactions to relatives, and how do professionals’ articulations of their respective positions inform their interactions with relatives?	Two cases constructed on the basis of 21 semi-structured interviews and a field study.	21 interviews with physicians, nurses, patients and their relatives.	Case 1: Relatives were involved in the sense that professionals appointed them with a low hierarchical position to care depending on what professionals considered best within the limits of the doxical values and logical function of the oncological clinic. Case 2: The neoliberal ideology and professional values form a dominant understanding of involvement that not only reject relatives’ first-person experiences but also constitutes harm. Relatives tended to experience distress, frustration and, at times, anger in their encounters when they felt obliged to get involved in psychiatric treatment.
[60]	To explore the elements that constitute supportive relationships and the meanings associated with them that can be the based for providing better, more focused support for young persons with mental health problems	Hermeneutic-phenomenological approach using in-depth individual interviews	14 young adults	Trusting the other to hold vulnerability safely—(a) power of holding, (b) trust as a moment of letting go, and (c) holding as a shared experience Flourishing in mutual participation – (a) mutual respect, (b) mutual disclosure, and (c) flourishing as possibility Acceptance in a felt togetherness—(a) acceptance that releases one from self-criticism, and (b) being-with as felt togetherness Feeling found and received—(a) feeling welcomed as significant to the other, and (b) feeling witnessed Feeling an attuned resonance—(a) tuned to the same frequency, (b) feeling touched in connecting with another, and (c) resonating with the other
[57]	To explore how service users experience barriers to help and assistance, and to determine the manner by which these barriers may influence their experiences of hope.	Thematic approach, using in-depth interviews	9 service users with MHSA problems	Battles with bureaucracy Distance, disempowerment, and de-individualization No clean slates.
[55]	Hvordan beskriver fagpersoner sitt samarbeid med beboerne for å styrke den enkelte beboers rolle? How do professionals describe their collaboration with residents to support and strengthen the person?	Phenomenological hermeneutic approach using multi stage focus group interviews.	6–8 professionals participated in 8 focus group interviews.	Four themes were developed, describing how professionals work to support the service users: Safety through relations, (relations as important foundation for feeling safe, safe relations developed through everyday life practices) Safety through the place (physical setting), Sense of pride through mastering (participation in settings like work, activities) Appraisal as a fertilizer for pride (focus on positive sides)
[62]	To explore how young people and parents experience collaboration with community-based mental health outreach team supporting the young people’s recovery processes	Phenomenological approach, using in-depth interviews to construct narratives	5 young adult service users and 4 parents	Two narratives about collaboration: Stories of the young people—(a) I need someone: To trust, Allows the time to build trust, Does not give up on me, Is there to help and like me, Takes initiative when I cannot; Is like a friend who can share something personal with me; (b) Be able to tell my stories or things to share, (c) Need to get help when I need, Do things together, Be here to help me when I need, Be able to meet when I need/want The stories of the parents—(a) Trust the person—The person is safe with my child, (b) Someone my child has a good relationship, (c) Help with things parents cannot, (d) Providing good help (flexible, suitable to my child’s needs), and (e) Need to consolidate multiple collaborative relations
